# Activity-based training with the Myosuit: a safety and feasibility study across diverse gait disorders

**DOI:** 10.1186/s12984-020-00765-4

**Published:** 2020-10-08

**Authors:** Florian Leander Haufe, Kai Schmidt, Jaime Enrique Duarte, Peter Wolf, Robert Riener, Michele Xiloyannis

**Affiliations:** 1 Sensory-Motor Systems (SMS) Lab, Institute of Robotics and Intelligent Systems (IRIS), ETH Zurich, Zurich, Switzerland; 2 MyoSwiss AG, Zurich, Switzerland; 3 Spinal Cord Injury Center, Balgrist University Hospital, Medical Faculty, University of Zurich, Zurich, Switzerland

**Keywords:** Training, Rehabilitation, Robot-assisted, Exoskeleton, Stroke, Spinal cord injury, Muscle dystrophy, Exosuit, Exomuscle

## Abstract

**Background:**

Physical activity is a recommended part of treatment for numerous neurological and neuromuscular disorders. Yet, many individuals with limited mobility are not able to meet the recommended activity levels. Lightweight, wearable robots like the Myosuit promise to facilitate functional ambulation and thereby physical activity. However, there is limited evidence of the safety and feasibility of training with such devices.

**Methods:**

Twelve participants with diverse motor disorders and the ability to walk for at least 10 m were enrolled in this uncontrolled case series study. The study protocol included five training sessions with a net training time of 45 min each. Primary outcomes were the feasibility of engaging in training with the Myosuit, the occurrence of adverse events, and participant retention. As secondary outcomes, we analyzed the walking speed using the 10-m Walk Test (10MWT) and for three participants, walking endurance using the 2-min Walk Tests.

**Results:**

Eight out of 12 participants completed the entire study protocol. Three participants withdrew from the study or were excluded for reasons unrelated to the study. One participant withdrew because of an unsafe feeling when walking with the Myosuit. No adverse events occurred during the study period for any of the participants and all scheduled trainings were completed. For five out of the eight participants that completed the full study, the walking speed when using the Myosuit was higher than to their baseline walking speed.

**Conclusions:**

Activity-based training with the Myosuit appears to be safe, feasible, and well-tolerated by individuals with diverse motor disorders.

## Background

Physical inactivity has been identified as the fourth leading risk factor for global mortality, only surpassed by hypertension, tobacco use, and hyperglycemia. To contain the risks associated with physical inactivity, the World Health Organization recommends that all adults engage in moderate intensity physical activity for at least 150 min each week [1].

Physical activity is also a recommended part of treatment for stroke patients [2], and for patients with incomplete spinal cord injury (SCI) [3], inherited neuropathies such Charcot–Marie–Tooth disease [4], heart failure [5], or chronic obstructive pulmonary disease [6]. These wide-ranging recommendations reflect the consistent association between increased physical activity and improved health-related quality of life (e.g. [7–9].).

In spite of the evident health benefits of physical activity, a large proportion of elderly individuals and individuals with limited mobility do not meet the recommended dose of physical activity in their daily lives [10]. In many of these cases, neurological, neuromuscular, or cardiovascular deficits prevent individuals from reaching moderate intensity levels during exercise. In some cases, they prohibit any voluntary exercise altogether.

To address this problem, various technological solutions like full-leg, rigid exoskeletons have been developed to assist overground mobility (e.g. [11–14].). The safety and feasibility of gait training with mobile exoskeletons has been evaluated in several longitudinal training studies for individuals with spinal cord injury [15–17] and hemiparesis following stroke [18, 19]. Rigid exoskeletons largely substitute the ambulatory function of severely affected or completely paralyzed individuals and enable them to walk. Electric motors are used to provide large assistive torques to the users’ leg joints via rigid linkages. This allows exoskeletons to support the majority of the users’ weight and advance the users’ legs without a major voluntary contribution from the leg muscles.

The typically large masses of mobile rigid exoskeletons increase limb inertia and thereby hinder walking at higher speeds. The highest walking speed achieved in previous training studies [15–19] was 0.67 m/s, while most speeds were as low as 0.1 m/s to 0.4 m/s. This is well below the speeds required to support individuals with residual mobility during moderate intensity exercise.

To assist this more capable section of the population, more lightweight wearable robots (also known as “exosuits”, “exomuscles” or “dermoskeletons”) have been proposed [20–24]. Unlike exoskeletons that act on all leg joints, these devices allow for—and require—the active participation of the user, and can (partially) assist walking over a larger range of speeds [20, 25]. Thereby, such wearable robots can provide assistance as needed for functional ambulation [25] while simultaneously modulating the cardiovascular load of their users according to exercise recommendations. For example, a soft robotic exosuit unilaterally acting on one ankle joint was shown to reduce the energy expenditure and interlimb asymmetry of individuals with hemiparesis following a stroke during walking at 0.5 to 1.3 m/s [20]. In previous work from our group, we demonstrated that a soft wearable robot actively supporting hip and knee extension, the Myosuit, enabled an individual with incomplete SCI to walk substantially faster when assisted [25]. More recently, we showed that this functional improvement also translates to an increase in exercise intensity and a momentary reduction of the energetic cost of transport [26]. In larger longitudinal studies, training with wearable robots acting on the hip joint was shown to result in an intrinsic reduction of the cost of transport for elderly individuals [23] and individuals following stroke [24].

Further work [27–29] has primarily focused on the functional effects of robotic movement assistance. A lightweight wearable robot that assists knee flexion and extension reduced momentary movement performance, but elicited larger intrinsic improvements after 2 weeks of exercise training than when training without the device in users with multiple sclerosis [27]. In larger randomized controlled trials with individuals post-stroke, training with a robotic knee brace was shown to result in only modest functional benefits that were comparable to the control group [28], while training with a hip exoskeleton resulted in more pronounced functional benefits [29].

While it is hard to synthesize common trends out of the limited studies available, it appears that the most pronounced improvements were so far achieved with devices that targeted a very specific motor deficit (e.g. ankle assistance [20] or hip assistance [24, 29] improved hemiparetic gait for individuals after stroke). Other findings with devices that bear promise to work for more diverse gait disorders were based on single-participant observations [22, 25, 26], and it remains unclear to what extend these results generalize to larger populations.

There is limited evidence of how a single wearable robot could effectively assist individuals with diverse neuromuscular and neurological gait disorders during exercise training. Such a wider applicability would be highly desirable considering that in everyday clinical life, patients present with a wide array of different conditions and functional deficits [30].

**Fig. 1:**
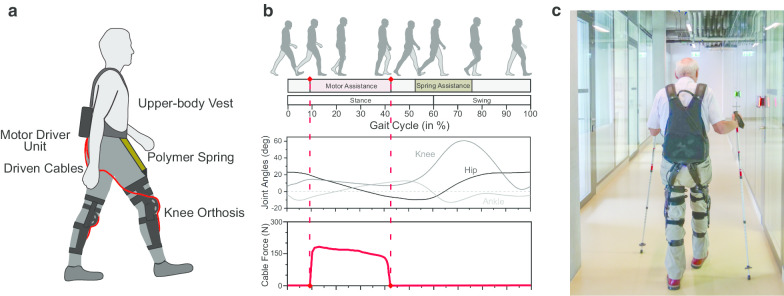
**a** Schematic drawing of the Myosuit. On each leg, an actuated cable is routed across the hip and knee joints and driven by electric motors contained in a backpack unit. **b** The motors tension the cables to apply forces assisting hip and knee extension against gravity during parts of the stance phase of walking. From terminal stance into swing, the springs assist hip flexion. **c** Exemplary picture of training with the Myosuit

We believe that the Myosuit is a promising device to assist the training of individuals with diverse gait disorders. The Myosuit assists walking in essential functions [31] by supporting the user’s bodyweight from weight acceptance through mid-stance and assisting swing initiation from terminal stance into early swing (see Fig. 1b). By working in parallel with the user’s hip and knee extensor muscles and exploiting natural synergies [32], the Myosuit supports the muscles with the largest contribution to bodyweight support in that phase of walking [33]. Training with the Myosuit can extend beyond step training and into areas of balance, strength, and coordination.

This support is packaged in a system that weighs only 5.5 kg which means little interference with the user’s movements [25]. The assistance level can be adjusted for each leg allowing for a high degree of personalization to the user’s training needs.

In this study, we investigated the safety and feasibility of assisting individuals across diverse neurological and neuromuscular gait disorders while training with the Myosuit. The study was designed as a longitudinal, uncontrolled case series with functional assessments (10MWT, for some participants 2minWT) in each session. The results from these functional assessments were primarily interpreted with respect to participant familiarization and tolerance to using the Myosuit. With the aim of maximizing the diversity among individual cases, we included participants that had a confirmed diagnosis of any pathology that leads to a weakness of the legs and were able to walk for at least 10 m without the assistance of a person (see Table 1). Internal tests prior to this study showed that these general inclusion criteria better captured the ability to use the Myosuit than for example individual muscle strength scores.

**Table 1:** Study eligibility criteria

Inclusion criteria	Exclusion criteria
Age between 18 and 80 years Body mass index of 30 kg/m^2^ or below Body height of 1.90 m or below Written informed consent Able to comply with all protocol requirements Confirmed diagnosis of a pathology that leads to a weakness of the legs Able to stand up from a chair and walk for 10 m without the assistance of a person	Guardianship/trusteeship Pregnant women Unstable cardiomyopathy Severe respiratory insufficiency Recent trauma

The present study is meant to inform the feasibility and design of a future randomized controlled trial in which the efficacy of Myosuit-assisted training can be systematically compared to conventional therapy.

## Methods

### Study participants and recruitment

Twelve participants were screened and recruited for this study (see Table 2) through referral from clinicians or word of mouth between September 2019 and February 2020 and participated in at least the initial training session after giving their informed consent. All participants were naïve, first-time users of wearable robotic technology. The study design and protocol were approved by the institutional review board of ETH Zurich (EK 2018-N-31) and classified as a non-interventional trial, thereby excluding it from trial registration.

**Table 2:** Study participant characteristics

ID	Symbol	Sex	Age (years)	Body height (cm)	Body mass (kg)	Case description	Time since event/diag	Self-reported walking level	Habitual mobility aids	Baseline 10MWT speed (m/s)	Mean daily step counts (1)
P1		f	43	164	48	Muscular atrophy due to Charcot–Marie–Tooth disease	n/a	Outdoor < 1 km	None	1.16	3′973
-		f				Multiple sclerosis Withdrew due to home clinic therapist advising against continuation				0.92	
-		m	55	178	74	Grade II brain tumor, right-sided hemiparesis. Excluded due to study-unrelated hospitalization				0.64	
-		m	27	175	69	Limb-girdle muscle dystrophy Withdrew because of unsafe feeling with the Myosuit				0.96	
P2		m	67	168	79	Motor-incomplete spinal cord injury at T7 (left) and T8 (right) (AIS D)	10.5 years	Outdoor < 1 km	Bilateral crutches or canes, wheelchair	0.53	3′891
P3		m	32	185	104	Sensorimotor-complete spinal cord injury at L4 (AIS A, detailed scores see Additional file 1: Fig. S3), cauda equina syndrome	8 years	Outdoor > 1 km	Bilateral crutches or canes, powered scooter	0.64	12′031
P4		m	54	186	67	Syringomyelia at T5 (AIS D, detailed scores see Additional file 1: Fig. S4)	18 years	Outdoor < 1 km	Bilateral crutches or canes	0.18	1′990
P5		m	72	164	72	Post-stroke left-sided hemiparesis	6 years	Outdoor < 1 km	Unilateral walking cane	0.45	5′637
–		m				Muscle dystrophy Excluded due to time constraints				0.68	
P6		m	63	177	73	Post-stroke right-sided hemiparesis	16 years	Outdoor > 1 km	None	1.70	9′011
P7		f	67	160	65	Spinal tumor at L1, L2, left-sided lower extremity paresis	24 years	Only indoor	Bilateral crutches or canes, wheelchair	0.38	349
P8		f	49	181	91	Surgical removal of m. psoas major, n. femoralis and n. obturatorius following muscle sarcoma	4.5 months	Outdoor > 1 km	Unilateral walking cane	0.92	4′288

*T* Thoracic spinal segment, *L* Lumbar spinal segment, *m.* musculus *n.* nervus

### Wearable robot

We used the Myosuit (MyoSwiss AG, Zurich, Switzerland) for this study.

The Myosuit is a wearable robot designed to assist across activities of daily life such as walking, standing, sitting transfers, or stair negotiation. It is comprised of a backpack-style motor driver unit, a textile upper body vest with a waist belt, and two polymer knee orthoses (see Fig. 1a). Two adjustable polymer springs frontally cross the hip joints to passively assist hip flexion. Two ultra-high-molecular-weight polyethylene cables are actively driven by two electric motors housed inside the backpack driver unit. The two cables are routed in textile guides across the user’s lower back and buttocks, one on each leg. They laterally cross the thighs and enter the knee orthoses in which they are frontally routed across the knee joints and anchored on the shin segments of the orthoses. This mechanical setup results in an underactuated coupling of hip and knee extension.

The electric motors are powered by an exchangeable Li-Ion battery, rendering the Myosuit completely untethered and autonomous. The motors generate linear forces of up to 230 N during normal walking and of up to 400 N during sit-to-stand transfers. This results in moments of 8 to 15 Nm across the knee joint and of 12 to 22 Nm with respect to the hip joint, assuming a mechanical transmission efficiency of 40% and anthropometric data from [34]. During walking, the cables were tensioned in parts of the stance phase (approx. between 10 and 40% of stride) to assist hip and knee extension against gravity (see Fig. 1b). The duration of assistance was individually adjusted for each leg and participant to maximize support but avoid undesirable locking towards the end of stance. During the assistive phase, the robotic controller modulated forces relative to the momentary knee angle (see Fig. 1b). Gait events and joint angles were estimated from inertial measurement unit data. The magnitude of the assistance was individually selected for each leg and each participant and kept constant for all assessments in the study (see Table 3). During other exercise modules, constant forces or force patterns designed to assist sit-to-stand transitions were applied. Myosuit assistance can be adjusted over 6 levels (0 to 5). The chosen setting acts as global scaling factor on the assistive forces provided by the suit, e.g. if the setting is 3, then 3/5 or 60% of the maximally available assistance during walking (230 N) is provided. Prototypes of the present device were described in more detail in previous work [21, 25].

**Table 3:** Myosuit assistance given as supporting force per unit of bodyweight (N/kg) and additional aids used by participants during 10MWTs and 2minWTs

ID	Symbol	Myosuit assistance left leg (N/kg)	Myosuit assistance right leg (N/kg)	Additional assistive devices
P1		1.0	1.9	–
P2		2.3	2.3	Bilateral crutches
P3		1.8	1.8	Bilateral crutches Bilateral ankle–foot orthoses
P4		2.1	2.1	Bilateral crutches Ankle–foot orthosis on right foot
P5		1.9	0.0	Cane on right side Ankle–foot orthosis on left foot
P6		1.9	1.9	–
P7		1.4	0	Bilateral crutches
P8		1.5	0	–

### Study protocol

#### General

All trainings were accompanied by a certified physiotherapist and a technical expert familiar with the Myosuit. Participants completed a total of five training sessions. Each session started with a 10-m Walk Test (10MWT), was then followed by a physiotherapy session of at least 45 min, and ended with a second 10MWT. In session 0, both 10MWTs were performed without the Myosuit. Three participants performed a 2-min Walk Test (2minWT) instead of the second 10MWT. The training sessions were set to last 45 min because this is the recommended and reimbursed length of an extended physiotherapy session in Switzerland [35]. After each session, the physiotherapists performed a brief examination of the body sites most susceptible to skin irritations, except for those that are very difficult to access (e.g. lower gluteal region, rear thighs).

During 10MWTs, participants were instructed to “walk as fast as safely possible from the start to the finish line”. The middle 6 m were considered for the calculation of walking speed. During 2minWTs, participants were instructed to “cover as much distance as possible within the next 2 min, always turning when reaching the ends of the (15.24 m (50 ft) long, straight [36]) walkway”. Habitual compensatory strategies and the use of additional personal assistive devices such as crutches, canes, or orthoses were intentionally allowed for to render the study more representative of typical exercise training, but kept consistent throughout the study (see Table 3). Training sessions were scheduled on a weekly basis, the participants’ schedules permitting.

### Training session 0

During the initial training session, therapists confirmed that the participants were able to stand up from a chair and walk for at least 10 m without assistance from a person. The participants’ baseline walking speed was calculated as the mean of two 10MWTs; one done at the beginning and one at the end of training. For participants who performed a 2minWT in lieu of the second 10MWT, the result from the first 10MWT was taken as-is. An exercise program for training was designed based on the individual participant’s functional status.

Following the assessments, participants were introduced to the Myosuit and assisted to don the device. They then engaged in the previously selected exercises for approximately 45 min of net training time before taking off the Myosuit. For three arbitrarily selected participants, a 2minWT without the Myosuit was performed at the end of training session 0.

### Interview after session 0

Two days after training session 0, participants were contacted by a physiotherapist and questioned in an unstructured, open-ended manner about their subjective experience with the Myosuit and about any adverse or positive effects of the training. Participants were asked if they experienced any pain during or after the training, if they felt fatigued after the training, and if they experienced delayed onset muscle soreness (DOMS) the day after training. Excessive fatigue and excessive DOMS were considered as adverse events. Other frequently discussed items included the regeneration after training, or any perceived influence on activities of daily living.

### Training session 1–4

At the beginning and end of each subsequent training session (1 to 4), a 10MWT with assistance from the Myosuit was performed. The participants engaged in the exercise program designed in training session 0 for 45 min. For those participants who performed a 2minWT without the Myosuit in training session 0, a 2minWT with assistance from the Myosuit was incorporated into the exercise program in lieu of the second 10MWT.

### Exercise modules

In addition to overground and treadmill walking training, various exercises targeting balance, strength, and combined functional tasks were included in the training program. Balance was practiced in different stances (one legged, parallel, step) and in combination with head and upper body rotations. To train leg muscle strength, the exercises included repeated unilateral eccentric and concentric hip extensor training, abduction movements, and bilateral eccentric and concentric knee extension exercises, similar to squats. Depending on their individual skill level, participants performed repeated sit-to-stand and stand-to-sit transitions and walked up and down stairs to combine these skills in relevant activities of daily life.

### Outcome measures

As primary outcomes we considered the feasibility of engaging in an exercise program with the Myosuit, the occurrence of adverse events, and participant retention. Feasibility was defined as the ability of the participants to complete their individual exercise program in all five training sessions, the 10MWTs and, if applicable, 2minWTs with the Myosuit.

As secondary outcome measures we considered the change in 10MWT walking speed and—for some participants—the change in 2minWT walking distance. We consider positive changes in these metrics indicative of familiarization and tolerance to using the Myosuit. In contrast, negative changes indicate a lack of familiarization and intolerance to using the Myosuit.

Additional measurements were taken for monitoring purposes and as exploration into potential confounders to the primary and secondary outcome measures. During all 10MWTs and 2minWTs, bidirectional audiovisual recordings were made. The participants’ heart rates were measured with wrist-worn fitness watches (Charge 3, Fitbit Inc., San Francisco, USA) and their perceived exertion was assessed using the Borg Scale [37]. The participants were asked to wear the fitness watches between training session during their daily activities to obtain a mean step count estimate representative of their activity level. The watches were linked to anonymized accounts to safeguard the participants’ privacy.

Many of these outcomes serve as exploratory variables to inform a future larger randomized controlled trial in which the efficacy of Myosuit-assisted training will be systematically compared to conventional therapy.

## Results

### Participant retention

Eight out of 12 enrolled participants reached the study endpoint and completed all five training sessions (see Table 2) for a total of 45 training sessions. One participant withdrew from the study because he did not feel safe with the Myosuit.

Three other participants withdrew or were excluded for reasons unrelated to the study. For one participant, the treating therapist at the home clinic advised against study continuation for undisclosed reasons. One participant could not attend subsequent sessions due to time constraints. For one participant, a routine check-up, unrelated to our study, revealed progressive growth of a pre-existing brain tumor that required immediate in-hospital treatment and precluded further participation in the study.

### Feasibility and safety

All eight participants who reached the study endpoint were able to complete their exercise program and the scheduled assessments in all training sessions (10MWT and in some cases, 2minWT). The remaining four participants were able to complete all scheduled assessments up until the point of withdrawal or exclusion from the study. No adverse events occurred during study sessions. Participant P4 reported moderate pain radiating along the sciatic nerve right after the training session, a condition that he reports to regularly experience also outside the study. All other participant experienced no pain after the training. No participant experienced excessive fatigue after the training. Five out of eight participants reported moderate DOMS on the days following the training sessions. The physiotherapists’ examination of skin contact sites after the trainings did not reveal any skin damage or bruises.

### Self-reported effects of training

All participants reported normal regeneration after training with the Myosuit. Some participants felt they slept better than usual the night after training, and some reported to generally feel more active and perform everyday movements more consciously. Other participants felt no effects following the training.

### Walking speed (10MWT)

**Fig. 2.**
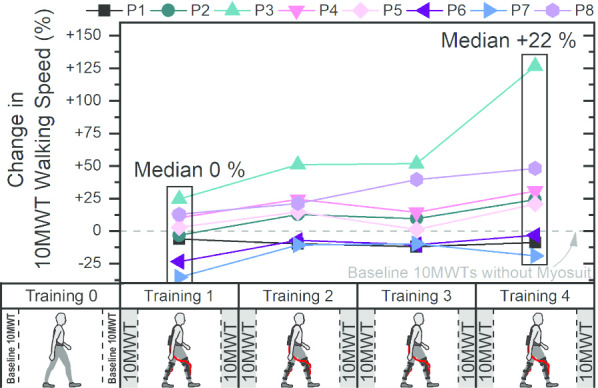
10MWT walking speed measured in trainings 1 to 4 with Myosuit assistance, relative to baseline 10MWT speed measured without the Myosuit in training 0. During the training blocks, the participants completed an individualized program comprising walking, balance and strength exercises. 10MWTs were performed at the beginning and end of training session, except for P6, P7 and P8, where only one 10MWT was performed at the beginning of the session and a 2minWT in place of the second 10MWT towards the end of the session (see also Fig. 3). Data points and baseline are calculated as the mean of the two 10MWTs during the respective session, and as the result from only the first 10MWT for P6, P7 and P8

From training session 2 onwards, five participants improved their walking speed (see Fig. 2). For three participants, the walking speeds were as fast as or slower than at baseline without the Myosuit.

The median walking speed was as fast as at baseline without the Myosuit during the first 10MWTs with the Myosuit (0% change, see Fig. 2). By training session 4, the median walking speed during 10MWTs was 22% faster than at baseline. Absolute changes in 10MWT walking speed in session 4 compared to baseline ranged from − 0.1 to 0.82 m/s.

### Walking endurance (2minWT)

**Fig. 3:**
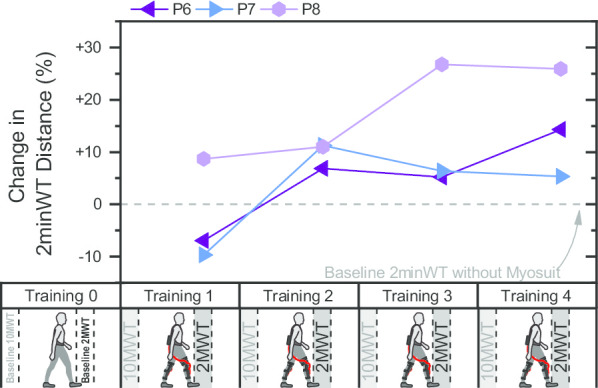
Distance covered in 2minWT during trainings 1 to 4 with Myosuit assistance, relative to baseline 2minWT distance measured without the Myosuit in training 0. Instead of the second 10MWT in training sessions 1 to 4, the last three participants performed a 2minWT during each training session

Three participants completed additional 2minWT during their trainings. For two out of these three participants their walking distance in the first 2minWT with the Myosuit was shorter than at baseline (training 1, see Fig. 3). All three participants were able to cover a longer distance with the Myosuit than at baseline from training session 2 onwards.

### Heart rate and perceived exertion

The participants’ heart rates presented with a large variability over a range of 66 bpm to 121 bpm at the end of 10MWTs (see Additional file 1: Figure S1) and 89 bpm to 141 bpm at the end of 2minWTs. The perceived exertion of participants measured with the Borg Scale ranged from 6 to 16 after both 10MWTs (see Additional file 1: Figure S2) and 2minWTs.

## Discussion

### Training with the Myosuit is feasible

Individuals with diverse neurological and neuromuscular motor disorders and substantially different baseline walking capacity (range in walking speed of 0.18 to 1.7 m/s, see Table 2) successfully engaged in Myosuit-assisted training (see Additional file 2).

Eight out of 12 enrolled participants reached the study endpoint. This retention rate is slightly higher than the ones found in previous studies with community-residing participants and rigid exoskeletons, where only 54% [15] and 50% [17] of the participants completed the entire protocol. These previous studies included more sessions and targeted more severely affected populations than our work. In a study with a more lightweight exoskeleton for partial movement assistance of the knee, 80% of participants completed the protocol [27].

Three out of the four participants who did not reach the current study’s endpoint withdrew or were excluded due to reasons unrelated to the study, e.g. time constraints or unrelated medical events. We speculate that this relatively high proportion of study-unrelated drop-outs can at least in parts be attributed to the present outpatient setting. The habitual environment of individuals could be an important factor for access and adherence, evidenced further in the fact that even very complex multi-visit studies with inpatient participants reported complete participant retention [16, 18]. We expect that future studies in which the Myosuit is used in inpatient settings might also show higher participant retention.

The current, relatively high retention rate is indicative of a good participant tolerance towards Myosuit-assisted training. It might further indicate that the participants’ expectations were met when training with the Myosuit, as also evidenced in the predominantly positive self-reported effects of Myosuit training. The unstructured format of our post-training interviews did not allow for a systematic analysis of these effects, though. In addition, our recruitment strategy of using referral from clinicians and word of mouth might have resulted in a study population with a favorably biased attitude towards robot-assisted training. Prospective participants willingly invested time and effort to travel to the study site and participate in at least the initial training session, likely because they expected to benefit from the Myosuit or were curious to test it.

### Training with the Myosuit is safe

No adverse events or adverse effects were reported during or after the more than 40 training sessions in which a variety of training exercises were performed (see “Exercise modules”). This shows that the protocol and exercise programs used in this study were safe, and that the Myosuit was well-tolerated by the diverse study population.

The only participant that withdrew from the study for study-related reasons, an individual with limb-girdle muscle dystrophy (see Table 2), reported feeling unsafe with the Myosuit. We attribute this feeling to an incompatibility between the participant’s pronounced compensatory strategy and the Myosuit assistance during walking that resulted in an undesirable trunk rotation during stance phase. Since this participant reported a history of falls, psychological factors might have added to the feeling of insecurity when walking with the Myosuit. This observation suggests that the perceived safety and feasibility of Myosuit training might decrease with an increasing departure from unimpaired gait kinematics.

The moderate DOMS reported by five participants might be attributed to elevated training intensity, and as such, could be desirable within reasonable limits.

### Individuals need time to adapt to the Myosuit

Due to the limited number of cases in our study, we interpret the positive trend observed in 10MWT and 2minWT performance primarily as additional evidence towards the safety and tolerance of Myosuit training, and not with regard to the clinical meaningfulness of improvements. In addition, a more individual interpretation of the assistive effects of the Myosuit is also limited by the lack of detailed muscle strength and sensory scores for our study participants (except P3 and P4, see Additional file 1: Figures S3 and S4).

The observed increase in 10MWT and 2minWT performance might originate from a motor adaptation process. In unpublished results from a concurrent study, we have seen that unimpaired participants needed a period of approximately 10 min of continuous walking to adapt to the assistance from the Myosuit. Similarly, participants in the present study could have perceived the Myosuit’s assistance as a disturbance initially as they lacked an internal model of assisted walking [38]. Previous research showed that the ability of stroke survivors to form such internal models is impaired [39], suggesting that individuals with a motor disorder might need a longer adaptation period than unimpaired individuals. One can speculate that for example the pronounced increase in 10MWT walking speed of participant P3 is at least in parts explained by an ongoing motor adaptation and familiarization process. This is also because the well-preserved leg muscle strength of P3 (hip extension muscle function grade 4 (left) and 5 (right), bilateral knee extension grade 5, see Additional file 1: Figure S3) would in principal not suggest P3 as prime candidate to benefit from Myosuit assistance.

Based on our findings, an initial decrease in performance for the first 90 min of exposure time (e.g. 2 training session a 45 min) might have to be expected for most users. An open area of research is the design of familiarization protocols that minimize the time required to adapt to the Myosuit’s assistance.

Two out of the three individuals whose 10MWT speed with the Myosuit was persistently slower than at baseline, P1 and P6, reported to not use any walking aids in everyday life, and presented with baseline walking speeds of more than 1 m/s. Thus, P1 and P6 likely did not require—and hence, might not have been able to profit from—robotic gait assistance, similar to unimpaired individuals. Hence, the habitual use of walking aids could be an effective additional criterion to identify individuals who potentially profit from Myosuit assistance. Their reduced performance might in parts be related to the moderate added weight by the Myosuit of 5.5 kg. Alternatively, the level of Myosuit assistance might have been chosen too high for these more capable individuals during the tuning heuristic used in this study (see Additional file 1). This would underline the persistent need for automated, non-subjective algorithms to adjust and personalize assistance for diverse users.

### Age and activity might confound training efficacy

**Fig. 4:**
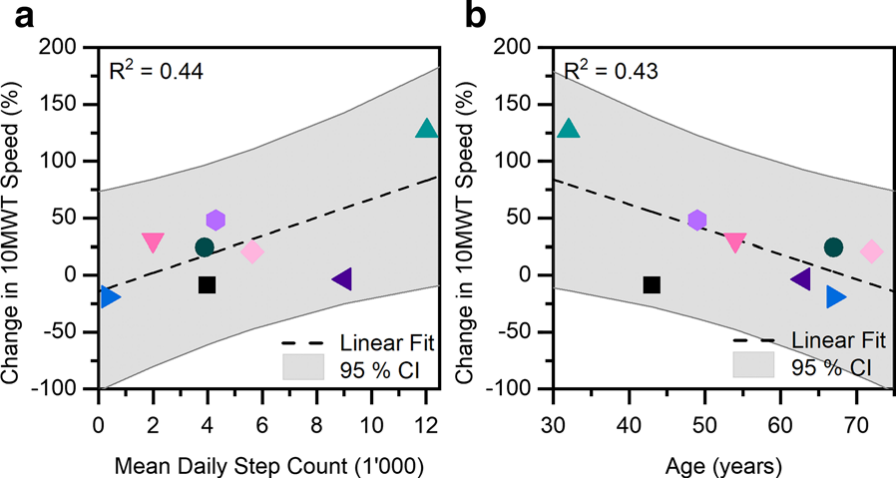
**a** Mean daily step count was moderately positively correlated with the observed change in 10MWT walking speed. The number of daily steps was recorded with a wrist-worn step counter during daily activities between training sessions. **b** The participants’ age was moderately negatively correlated with the observed change in 10MWT walking speed. P3 (green triangle) showed the largest increase in 10MWT speed, was the most active during daily life, and the youngest of all participants

Two independent simple linear regression analyses between our supporting outcome metrics and the observed change in 10MWT walking speed indicate a moderately positive correlation with mean daily step count (t(6) = 2.2, p = 0.07, R^2^ = 0.44, see Fig. 4a) and a moderately negative correlation with age (t(6) = -2.1, p = 0.08, R^2^ = 0.43, Fig. 4b). While these potential correlations seem reasonable—more active and younger individuals might achieve a larger improvement in 10MWT walking speed—, the limited size of our study population and resulting lack of statistical evidence mandate caution during further interpretation. In particular participant P3 stands out in terms of age, daily step count, and the observed change in 10MWT walking speed, and thereby substantially affects the regression results. Future controlled trials should consider matching age and the habitual activity level between study groups.

### Next steps to assess training efficacy

Building on the results from this study, the next step is to evaluate the efficacy of Myosuit-assisted exercise training compared to conventional exercise training. Future work should clarify to what extent the observed positive trend in walking performance originates from motor adaptation to the Myosuit, constitutes an improvement of intrinsic motor performance, or is a psychologically-driven placebo effect. Further, additional assessments like the Berg Balance Score will help us analyze the effects of Myosuit training beyond walking function. Such quantitative assessments should be supported by patient-reported outcome measures through a more systematic use of questionnaires. A cross-over study design, as for example in [27], might help to efficiently analyze the available participant population.

## Conclusions

Activity-based training with the Myosuit is safe, feasible and well-tolerated by individuals with diverse neurological and neuromuscular motor disorders.

This first clinical test with the Myosuit provides evidence that a larger, randomized controlled trial with one Myosuit-assisted and one conventional training arm would be feasible, even when including a diverse population. Our study results also suggest a positive effect on walking speed and endurance in basically ambulatory subjects when supported by the Myosuit.

## Supplementary information

**Additional file 1: Figure S1.** Mean Heart Rate after completion of 10MWTs across training session 1 to 4. The mean heart rate is calculated as the mean of the heart rates after the two 10MWTs in each session, except for P6, P7 and P8, where only one 10MWT was performed. **Figure S2.** Mean Borg Scale Rating of 10MWTs across training session 1 to 4. The mean rating is calculated as the mean of the ratings of the two 10MWTs in each session, except for P6, P7 and P8, where only one 10MWT was performed. **Figure S3.** Detailed key muscle strength and sensory scores (light touch and pin prick) for participant P3. The form has been reproduced by the authors without any personal information to safeguard the participants privacy. **Figure S4. **Detailed key muscle strength and sensory scores (light touch and pin prick) for participant P4. The form has been reproduced by the authors without any personal information to safeguard the participants privacy.

**Additional file 2.** Video file presenting exemplary exercises and assessments (10MWT and 2minWT) during activity-based training with the Myosuit.

## Data Availability

All the data relevant to this study are presented within the manuscript.

## Abbreviations

SCI: Spinal cord injury
10MWT: 10-M Walking Test
2minWT: 2-Min Walk Test
SEM: Standard error of mean
