# Supplementary material for: Activity-based training with the Myosuit: a safety and feasibility study across diverse gait disorders
Source: J Neuroeng Rehabil. 2020 Oct 8;17:135. doi: 10.1186/s12984-020-00765-4 (PMC7545901; doi:10.1186/s12984-020-00765-4)
Supplement: Supplementary file 1 — Additional file 1: Figure S1. Mean Heart Rate after completion of 10MWTs across training session 1 to 4. The mean heart rate is calculated as the mean of the heart rates after the two 10MWTs in each session, except for P6, P7 and P8, where only one 10MWT was performed. Figure S2. Mean Borg Scale Rating of 10MWTs across training session 1 to 4. The mean rating is calculated as the mean of the ratings of the two 10MWTs in each session, except for P6, P7 and P8, where only one 10MWT was performed. Figure S3. Detailed key muscle strength and sensory scores (light touch and pin prick) for participant P3. The form has been reproduced by the authors without any personal information to safeguard the participants privacy. Figure S4. Detailed key muscle strength and sensory scores (light touch and pin prick) for participant P4. The form has been reproduced by the authors without any personal information to safeguard the participants privacy. [file 12984_2020_765_MOESM1_ESM.docx]

Additional file 1

Supporting Material to *“Activity-based training with the Myosuit: a safety and feasibility study across diverse gait disorders”*

# Individual selection of Myosuit assistance settings

The individual selection of assistive forces was performed following the below heuristic:

1. Participants started to walk with uni- or bilateral assistance from the Myosuit depending on the laterality of their leg weakness. Forces were set to 80 % of the maximum (230 N) and the timing of force application was at the default (fixed delay after heelstrike to approx. 16 degrees of hip extension) during the first steps of walking.
2. If therapists observed that participants got locked with an extended leg during late stance and could not flex and advance their leg as desired, the duration of force application was manually reduced by the therapist. If participants felt insufficient support during mid- to late stance, the therapist manually increased the duration of force application.
3. The therapist iteratively changed the force magnitude from 80 % to 60 % and upon discretion, to lower settings, to determine the setting that resulted in the subjectively best movement performance. In parallel, participants were questioned about their preferred setting and the final magnitude was determined as aggregate of the two.
4. If necessary, the tension of the polymer hip springs was increased to provide more flexion assistance during swing initiation.

# Heart Rate during 10MWTs


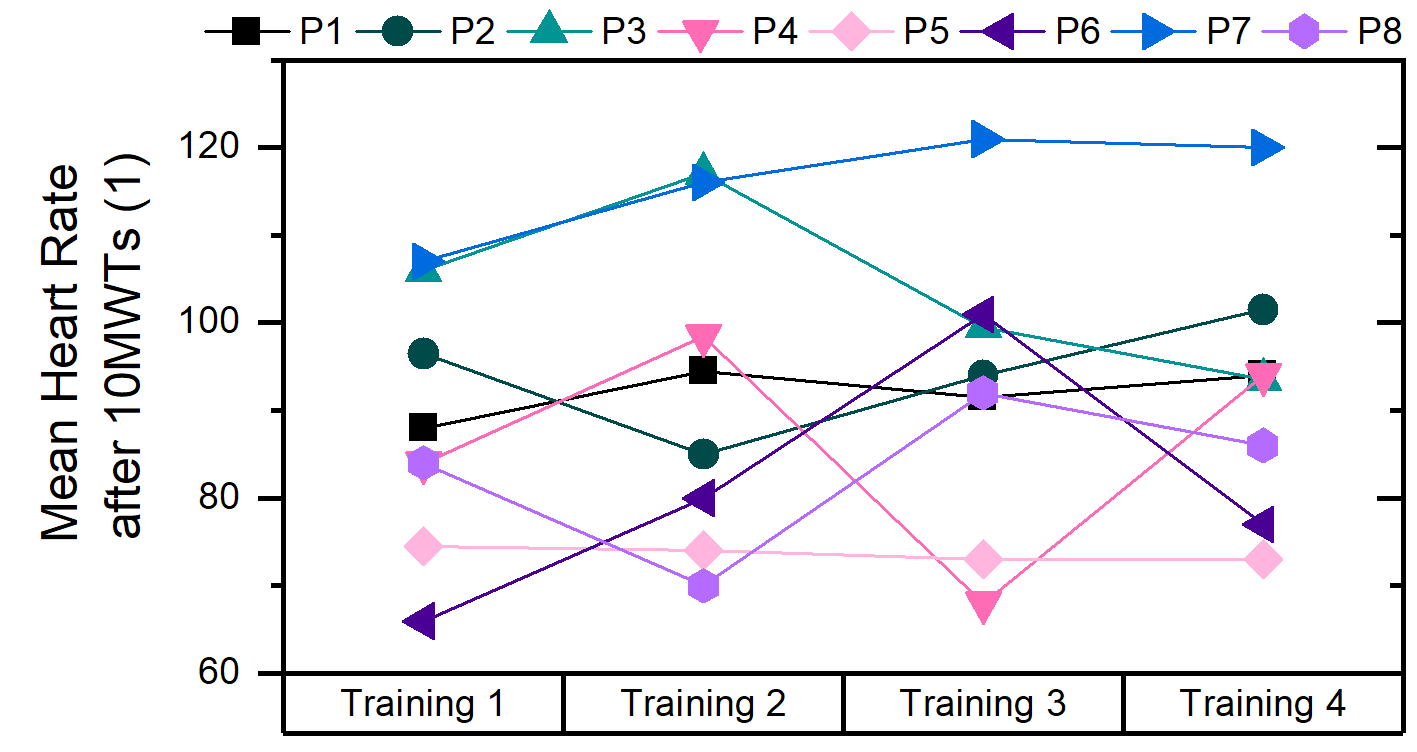


Figure S1. Mean Rate after completion of 10MWTs across training session 1 to 4. The mean heart rate is calculated as the mean of the heart rates after the two 10MWTs in each session, except for P6, P7 and P8, where only one 10MWT was performed.

# Borg Scale Ratings during 10MWTs


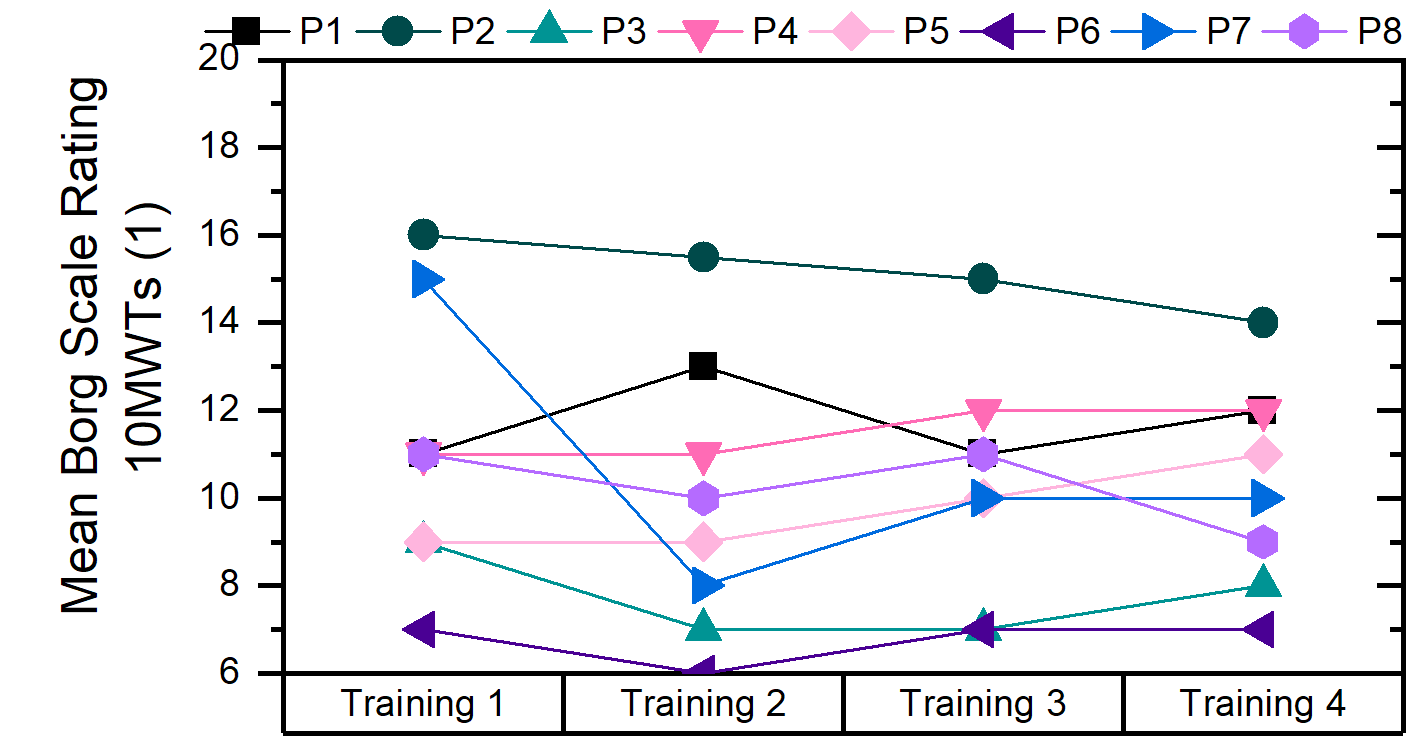


Figure S2. Mean Borg Scale Rating of 10MWTs across training session 1 to 4. The mean rating is calculated as the mean of the ratings of the two 10MWTs in each session, except for P6, P7 and P8, where only one 10MWT was performed.

# Detailed Muscle Strength and Sensory Function Participant P3


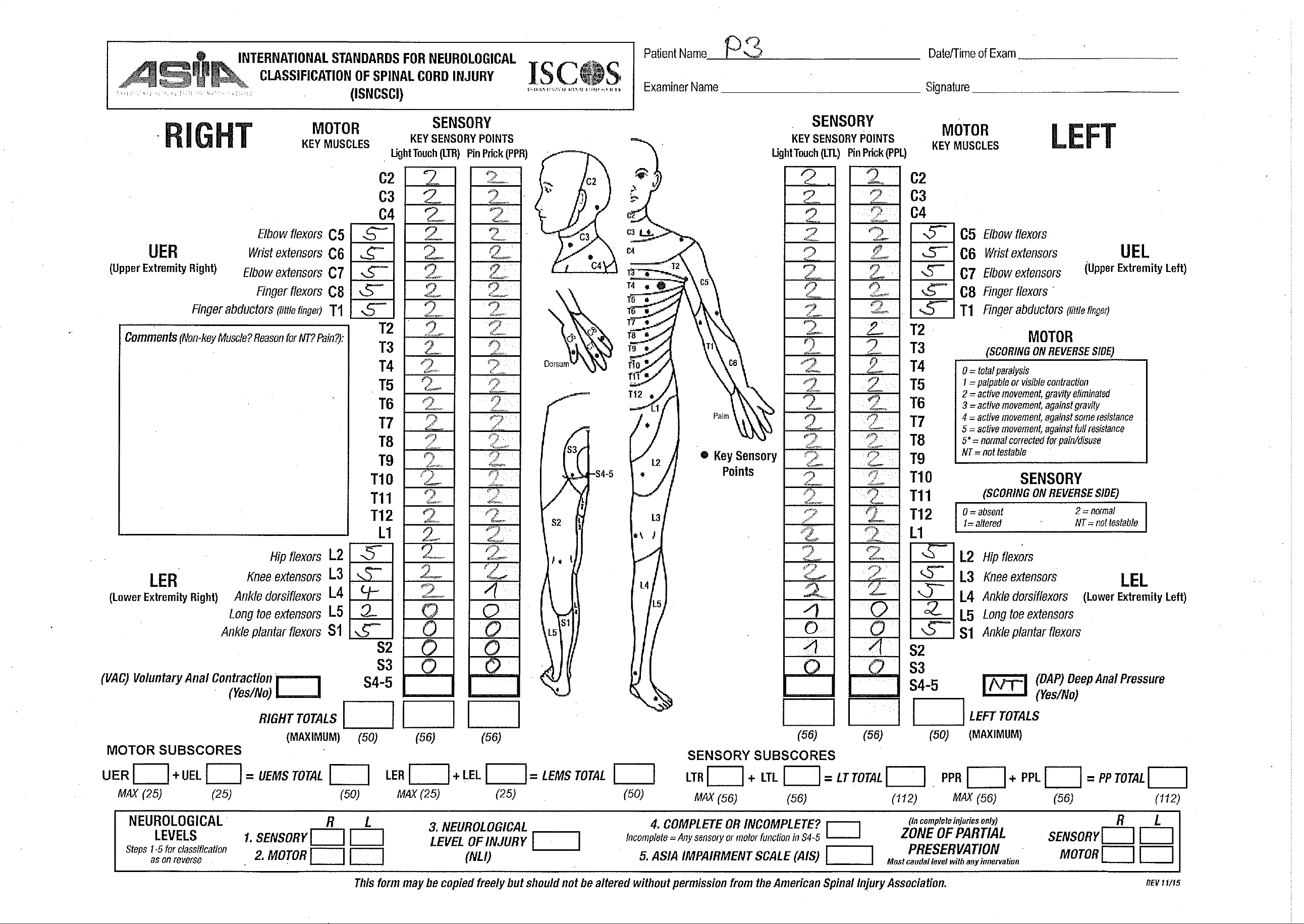


Figure S3. Detailed key muscle strength and sensory scores (light touch and pin prick) for participant P3. The form has been reproduced by the authors without any personal information to safeguard the participants privacy.

# Detailed Muscle Strength and Sensory Function Participant P4


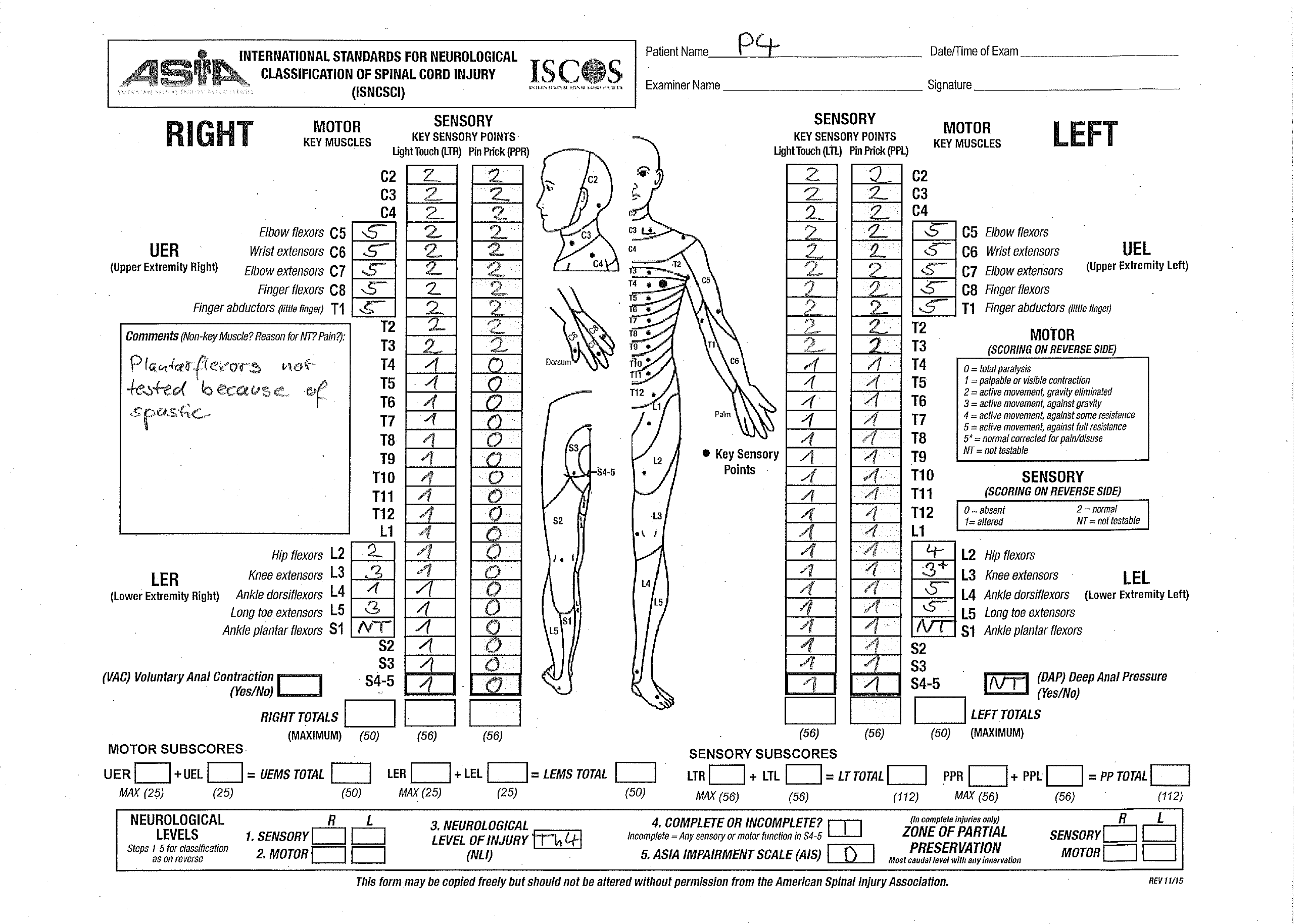


Figure S4. Detailed key muscle strength and sensory scores (light touch and pin prick) for participant P4. The form has been reproduced by the authors without any personal information to safeguard the participants privacy.
